# *In vitro *evaluation of marine-microorganism extracts for anti-viral activity

**DOI:** 10.1186/1743-422X-7-182

**Published:** 2010-08-07

**Authors:** Jarred Yasuhara-Bell, Yongbo Yang, Russell Barlow, Hank Trapido-Rosenthal, Yuanan Lu

**Affiliations:** 1 Department of Tropical Medicine, Medical Microbiology and Pharmacology, John A. Burns School of Medicine, University of Hawaii at Manoa, 651 Ilalo Street, BSB Suite 320, Honolulu, HI, 96813, USA; 2 Department of Public Health Sciences, John A. Burns School of Medicine, University of Hawaii at Manoa, 1960 East West Road, BIOMED D104K, Honolulu, HI, 96822, USA; 3 Center for Marine Microbial Ecology and Diversity, 1680 East West Road, POST 105 University of Hawaii at Manoa, Honolulu, HI, 96822, USA

## Abstract

Viral-induced infectious diseases represent a major health threat and their control remains an unachieved goal, due in part to the limited availability of effective anti-viral drugs and measures. The use of natural products in drug manufacturing is an ancient and well-established practice. Marine organisms are known producers of pharmacological and anti-viral agents. In this study, a total of 20 extracts from marine microorganisms were evaluated for their antiviral activity. These extracts were tested against two mammalian viruses, herpes simplex virus (HSV-1) and vesicular stomatitis virus (VSV), using Vero cells as the cell culture system, and two marine virus counterparts, channel catfish virus (CCV) and snakehead rhabdovirus (SHRV), in their respective cell cultures (CCO and EPC). Evaluation of these extracts demonstrated that some possess antiviral potential. In sum, extracts 162M(4), 258M(1), 298M(4), 313(2), 331M(2), 367M(1) and 397(1) appear to be effective broad-spectrum antivirals with potential uses as prophylactic agents to prevent infection, as evident by their highly inhibitive effects against both virus types. Extract 313(2) shows the most potential in that it showed significantly high inhibition across all tested viruses. The samples tested in this study were crude extracts; therefore the development of antiviral application of the few potential extracts is dependent on future studies focused on the isolation of the active elements contained in these extracts.

## Background

Viruses cause many important diseases in humans, with viral-induced emerging and re-emerging infectious diseases representing a major health threat to the public. In addition, viruses can also infect livestock and marine species, causing huge losses of many vertebrate food species. Effective control of viral infection and disease has remained an unachieved goal, due to virus' intracellular replicative nature and readily mutating genome, as well as the limited availability of anti-viral drugs and measures.

The use of natural products in the manufacturing of drugs is an ancient and well-established practice that has yielded such familiar products as morphine, digitalis, penicillin, and aspirin [1]. Natural products derived from terrestrial and marine kingdoms represent an inexhaustible source of compounds with promising antiviral action, not only for the great number of species found in these kingdoms with unexplored pharmacological activities, but mainly for the variety of synthesized metabolites. In relation to infectious diseases, the exploration of the marine environment represents a promising strategy in the search for active compounds, whereas there is a need for new medicines, due to the appearance of resistance to available treatments in many microorganisms, specifically concerning antifungal, antiprotozoal, antibacterial and antiviral activities.

The marine environment represents approximately half of the global biodiversity and could provide unlimited biological resources for the production of therapeutic drugs [1-3]. Almost all forms of life in the marine environment (e.g. algae, sponges, corals, ascidians) have been investigated for their natural product content [4]. Ecological pressures, such as competition for space, predation, symbiosis and tide variations, throughout thousands of years, originated the biosynthesis of complex secondary metabolites by these organisms, which in turn, allowed their adaptation to a competitive and hostile environment [3].

The first serious work on marine organisms started only 50 years ago. In the following 50 years, marine organisms (algae, invertebrates and microbes) have provided key structures and compounds that proved their potential for industrial development as cosmetics, nutritional supplements, fine chemicals, agrochemicals and therapeutic agents for a variety of diseases. Some examples of commercially available marine bioproducts that have been developed include: a) Ara-A (vidarabine) and Ara-C (cytarabine) (antiviral drugs) derived from the sponge *Tethya cripta*; b) Okadaic acid and Manoalide (molecular probes) from Dinoflagellate and the sponge *Luffariella variabilis*, respectively; c) Green Fluorescent Protein (GFP, Reporter gene) from the jellyfish *Aequora victoria*; d) Phycoerythrin (conjugated antibodies) used in Enzyme-Linked ImmunoSorbent Assays (ELISA) and flow cytometry from red algae and; e) Pseudopterosins (additives in skin crèmes) from the soft coral *Pseudopterogorgia elizabethae *[1]. As a result, important pharmacological and therapeutic products are currently being obtained and actively sought from the ocean [1,2,4-21].

The current antiviral drug armamentarium comprises over 40 compounds that have been officially approved for clinical use, with at least half of them being used to treat HIV infection [1,3,17]. Marine antiviral agents (MAVAs) [22] can be used for the biological control of human enteropathogenic virus contamination and disease transmission in sewage-polluted waters, as chemotherapy for viral diseases of humans and lower animals, as well as the biological control of viral diseases of marine animals. The seeding of MAVAs under natural conditions, or when marine mammals are kept in captivity for various uses, could control viral disease transmission within these select populations. It is clear that the marine environment will play a vital role in the future development and trials of anti-infective drugs.

Within the Environmental Health Laboratory at the University of Hawai'i at Manoa, four representative viruses isolated from mammal-and marine-animal species were collected and prepared. In addition, a cell line bank was established, comprising over 150 cell lines derived from various organs and tissues of different animal species. Also, over 2,000 unpurified crude extracts from a variety of marine organisms, including sponges, bacteria and algae, have been prepared in Dr. Thomas Hemscheidt's laboratory at the University of Hawai'i at Manoa. These compounds and extracts were initially being tested for anti-bacterial and anti-tumor activities. The purpose of this study was to establish an in vitro model to screen marine extracts for antiviral activity and to evaluate 20 marine extracts for their antiviral potential, with a long-term goal of discovering new marine compounds to be used as potential antiviral drug candidates.

## Methods

### Cell Cultures

Readily available cell cultures essential for supporting viral infectivity of the test viruses (Table 1) were used in this study. Green African monkey kidney (Vero) cells (ATCC^®^, Manassas, VA, Cat. No. CCL-81™) and Epithelioma papulosum cyprini (EPC), carp skin cells (ATCC^®^, Manassas, VA, Cat. No. CRL-2872™) were grown with Eagle's minimal essential medium (MEM) (Sigma-Aldrich, St. Louis, MO) supplemented with 10% heat-inactivated bovine calf serum (BCS) (HyClone, Logan, UT) and 1% GPS solution (100 U/mL penicillin, 100 μg/mL streptomycin sulfate, and 4 mM L-glutamine: Sigma-Aldrich, St. Louis, MO) at 37°C with humidified 5.0% CO_2 _and at room temperature (23 ± 1°C) under normal atmospheric conditions, respectively. Chanel catfish ovary (CCO) cells (ATCC^®^, Manassas, VA, Cat. No. CRL-2772™) were grown with high-glucose Dulbecco's modified Eagle's medium (DMEM) (Sigma-Aldrich, St. Louis, MO) supplemented with 10% heat-inactivated standard fetal bovine serum (FBS) (HyClone, Logan, UT) and 1% GPS solution at room temperature.

**Table 1:** Cell culture systems and representative viruses.

Cells		Virus		
Name	Species of Origin	Susceptable viruses	Viral Family	Host
**Vero**	African Green Monkey kidney epithelial cells	**HSV-1 **(herpes simplex virus type 1) **VSV **(vesicular stomatitis virus)	*Herpesviridae* *Rhabdoviridae*	Mammalian
**EPC**	Cyprinis carp skin	**SHRV **(snakehead rhabdovirus)	*Herpesviridae*	Marine
**CCO**	Channel catfish ovary	**CCV **(channel catfish virus)	*Rhabdoviridae*	

Cells were subcultured at a 1:3 ratio every 3-4 days. Briefly, media from TC-75 cm^2 ^flasks were collected and centrifuged at 3000 rpm for 5 minutes. Meanwhile, 5.5 ml/flask of a trypsin-versine solution (10 ml 10 × Trypsin (Sigma-Aldrich, St. Louis, MO) in 90 ml pre-sterilized versine (EDTA) solution) was added to detach the cell monolayer [23]. Following cell detachment, cleaned medium was added back into the flasks to neutralize the trypsin activity. The contents of the flasks were then removed and centrifuged at 1000 rpm for 5 minute. Following centrifugation, supernatant was removed and new growth medium was used to resuspend the cells. Cells, split 1:3, were placed back into flasks and total media volume was brought up to 10 ml/flask. Flasks were then placed back into their respective incubators and monitored daily. The pH of the medium was monitored and adjusted to 7-7.5 using HEPES buffer (Mediatech, Herndon, VA) or 7.5% w/v NaHCO_3 _(Mediatech, Herndon, VA).

### Viruses

The viral isolates used in this study (Table 1) are available in the laboratory and methodologies for their replication and purification, as well as quantitative infection assays, have been established and routinely used [24]. These representative indicator viruses were propagated and quantified as viral stocks for this study. Briefly, cells were grown and seeded into TC-75 cm^2 ^flasks, as previously described, so that an approximately 90% cell monolayer formed in 24 hours. All medium was removed from the flask and 250 μl of previously made virus stock was mixed with 2 ml of serum-free medium and added into the flask to infect the cells. The flasks were incubated for 1 hour and then inoculum was removed. Cells were washed twice with serum-free medium and then 10 ml of medium supplemented with 5% serum was added into the flask. The flasks were then incubated at the optimal temperature for viral replication, until the visual appearance of approximately 90% cytopathic effects (CPE) (rounding of cells, loss of contact inhibition and cell death), after which the flasks were stored at -80°C for 24 hours. Following two cycles of the freeze-thaw, the contents of the flasks were completely harvested and centrifuged at 1000 rpm for 5 minutes to remove all cellular debris. Supernatant was then collected and aliquots of 0.5 ml/tube were stored long-term at -80°C or short-term at -20°C. Viral titers were determined using plaque assays, as described below.

### Extracts

Twenty marine-microorganism extracts were tested for their antiviral activities in this study. These extracts were provided from Dr. Thomas Hemscheidt's laboratory at the University of Hawai'i at Manoa (Table 2). Microbial colonies were collected from sites around the Hawaiian Islands and various sites in the open ocean. Briefly, cultures were isolated, made axenic, identified by 16 s ribosomal DNA (rDNA) PCR, classified, and submitted for culturing. Upon receipt, each culture was given a Center for Marine Microbial Ecology and Diversity (CMMED) number and cryogenically frozen in quartet (if possible). An example of a CMMED# is as follows: 288 (1), where the (1) denotes that this was the first grow out of this particular culture and subsequent grow outs of the same culture are denoted as (2), (3) etc. To harvest and extract marine bacteria, cultures were spun down and pelleted at 5,000 g for 18-20 min. The supernatant was then extracted with ethyl acetate and the pellet was extracted with 2:1 methylene chloride: 2-propanol. Cultures that had both the media/supernatant and pellet extracted are differentiated from one another by the addition of an M to the CMMED# to denote a media extraction (e.g. CMMED# 288 M (1)). To extract diatoms, cyanobacteria, etc., entire cultures skipped the harvesting and both the cells and media were extracted with ethyl acetate. Cultures that were extracted without pelleting were given an M on the extract number. Solvent was then removed via overnight speed vacuum. The samples were then dissolved in DMSO at a concentration of 100 mg/ml and then used for screening.

**Table 2:** Marine extracts and their antiviral effects.

Extract	Source	Herpesvirus		Rhabdovirus	
		Mammalian	Marine	Mammalian	Marine
		HSV-1	CCV	VSV	SHRV
**162M(4)**	Marine bacterium; unclassified	+++	+	+++	N/T
**185M(4)**	*Roseobacter sp*.	+	N/T	++	N/T
**219M(3)**	*Pseudoalteromonas sp*.	+	N/T	+++	N/T
**258M(1)**	Cyanobacterium; Blue-green algae	+++	N/T	+++	N/T
**298M(2)**	Marine bacterium; unclassified	+++	+++	+++	+
**312(2)**	Marine diatom; *cf. Odontella sp*.; Bacillariophyceae	+++	N/T	+++	N/T
**313(2)**	Marine diatom; *Amphora sp*.; Bacillariophyceae	++	+++	+++	+++
**328(2)**	Marine diatom; *cf. Odontella sp*.; Bacillariophyceae	+	N/T	+++	N/T
**331M(3)**	*Shewanella frigidmarina*	+	+++	-	+
**338(1)**	*Bacillus methanolicus*	-	N/T	+	N/T
**338M(1)**	*Bacillus methanolicus*	+	N/T	+	N/T
**367M(1)**	Marine bacterium; unclassified	+++	N/T	+++	N/T
**388(1)**	Marine bacterium; unclassified	-	++	+	-
**397(1)**	Marine bacterium; unclassified	-	++	+++	-
**397M(1)**	Marine bacterium; unclassified	N/T	+++	N/T	+
**438M(1)**	Marine bacterium; unclassified	++	N/T	-	-
**460(1)**	Marine bacterium; mixed	-	N/T	++	N/T
**475(1)**	Marine bacterium; unclassified	++	N/T	++	N/T
**476(1)**	Marine bacterium; Proteobacteria/Halomonas	++	N/T	+++	N/T
**491(1)**	Marine bacterium; unclassified	-	N/T	-	N/T
**495M(1)**	Marine bacterium; unclassified	++	+++	++	N/T

- = No meaningful inhibition (< 20%); + = Slight inhibition (≥ 20%); ++ = Moderate inhibition (≥ 50%); +++ = High inhibition (≥ 80%); N/T = not tested.

### Plaque Assay

Briefly, cells were cultured and then seeded into multi-well plates at density that would allow the formation of an approximately 90% monolayer in 24 hours. Once a confluent cell monolayer was formed, media from the wells was aspirated. Meanwhile, serial 10-fold dilutions of stock virus were made and 100 μl/well of each viral dilution were added to the plates. Plates were incubated for 1 hour, then inoculum from each well was completely removed and 2 ml/well of a 0.75% (w/v) methylcellulose overlay medium, containing 5% serum and 1% GPS solution, was added. Plates were then incubated for 3-4 days to allow viral plaque development. Viral plaques were visualized by the addition of 2 ml/well of crystal violet staining solution for at least 2 hours [25] and vigorous washing with tap water. Plaques were counted visually and the viral titer calculated as follows: Virus Titer (PFU/ml) = [# plaques counted × dilution factor'/amount of viral inoculum used (0.1 ml).

### Cytotoxicity Assay

Briefly, cells were maintained, as previously described, and then seeded into 96-well plates at a density that would allow the formation of a 90% monolayer in 24 hours. Once a confluent cell monolayer was observed, media from the wells was removed. Each extract was diluted in medium supplemented with 5% serum, with subsequent DMSO dilutions used as controls. For purposes of this study, four concentrations, including 100, 50, 25 and 12.5 μg/ml, were tested. Control dilutions of DMSO at 0.1%, 0.05%, 0.025% and 0.0125% were also included. Then, 200 μl/well of diluted extract and DMSO controls were added to the plates, at 4 wells/concentration, and then the plates were incubated for 3 days.

A Methylthiazol Tetrazolium (MTT) assay commonly used for cell proliferation was adopted to test for cell viability. In brief, following the 3-day incubation, 20 μl/well of MTT (VWR, West Chester, PA) was added to each plate. The plates were then incubated in a dark incubator for 2-4 hrs, with checking every 30 minutes for purple formazan crystal formation. Once proper formazan crystal formation was observed, the contents from the wells were completely aspirated. Immediately after, 100 μl/well of 100% DMSO was added to each plate and then incubated at room temperature on a mixer for 30 minutes. Absorbance at 570 nm was read on a microplate reader (Beckman Coulter AD 340C, Beckman Coulter, Fullerton, CA). Any extract producing a 10% or more reduction in cell viability was considered toxic.

### Viral Attachment/Entry Inhibition Assay

Cells at exponential growth phase were harvested and seeded into multi-well plates at densities that would allow the formation of an approximately 90% cell monolayer overnight. Marine extracts were diluted with serum-free medium to twice the effective safe concentrations, as determined by the cytotoxicity tests. Viruses were diluted in serum-free medium to optimum concentrations that would yield approximately 50-100 PFU/well, as determined by previous plaque assays. Then, 250 μl of each extract at twice the maximum nontoxic concentration (e.g., 200 μg/ml for those found to be nontoxic at 100 μg/ml) was mixed with an equal volume of the virus dilution. Positive controls were made by mixing 250 μl of virus dilution with 250 μl of serum-free medium with 0.2% DMSO, in order to yield a final DMSO concentration of 0.1%. These 500 μl virus/extract mixtures were pre-incubated for 1 hour, along with controls, and then assayed for viral infectivity using the optimized plaques assay protocols. Extracts producing a reduction in plaque formation were considered for further characterization. Antiviral effect of each extract was categorized as having no meaningful inhibition (< 20%), slight inhibition (≥ 20%), moderate inhibition (≥ 50%), or high inhibition (≥ 80%).

### Viral Replication Inhibition Assay

Test cells were seeded into TC-12.5 cm^2 ^flasks (BD Falcon, San Jose, CA) at a density that would allow the formation of an approximately 90% monolayer the next day. Marine extracts were diluted with medium containing 5% serum to their safe and effective concentrations, as determined by the cytotoxicity tests. Medium was completely aspirated from the flasks, and then the cell monolayer was briefly washed with DPBS, before infection with test virus at a multiplicity of infection (MOI) of 0.1. Following a 1-hr viral adsorption, all medium in the flask was removed and the flasks were washed twice with DPBS (Sigma-Aldrich, St. Louis, MO). Infected cultures were incubated with 2.5 ml/flask of diluted extract. Two flasks were tested per extract and these cultures were allowed to incubate for 3 days. Pictures were taken every 12 hrs using an inverted microscope equipped with a camera (Nikon Eclipse TE2000-U), starting at time zero, in order to track the progression of viral-induced CPE. To track viral progression, 200-μl samples of medium were taken from each flask, every 12 hours, and stored at -20°C until the end of the experiment. The viral titers of these samples were later determined by standard plaque assay, as previously described. Test extracts shown to produce a visually noticeable reduction in CPE, as well as a reduction in viral titer, were considered for further characterization.

### Data Analyses

Using OriginPro 8 (OriginLab Corporation, Northampton, MA), a one-way ANOVA was performed on the data to determine significance. The alpha value was set at 0.05 to yield a significance with > 95% confidence.

## Results

### Extract Cytotoxicity

To properly assess these marine extracts for antiviral activity, a set of experimental tests were performed to determine the safe and effective dose of these extracts to be used for each cell culture system. Experimental results revealed that extracts 298M(2), 313(2), 331M(3) and 438M(1) were toxic to Vero cells at a dose of 100 μg/ml, with 298M(2) definitively being the most toxic (P < 0.001), followed by 313(2), 331M(3) and 438M(1) (P < 0.05, P < 0.05 and P < 0.5, respectively) (Table 3). These four extracts also showed varied levels of cytotoxicity at a concentration of 50 μg/ml, although this apparent toxicity was far less, if not negligible, as compared to that observed at a concentration of 100 μg/ml. These observations are consistent with that observed visually through a microscope. To be safe, these three extracts were used at a concentration of 25 μg/ml in the latter experiments involving Vero cells. All other extracts were found to be nontoxic to Vero cells at all tested concentrations and were therefore used at 100 μg/ml in the latter experiments involving Vero cells.

**Table 3:** Summary of extract cytotoxicity.

Cells	Extract	Extract Concentration			
		12.5 mg/ml	25 mg/ml	50 mg/ml	100 mg/ml
**Vero**	**298M(2)**	-	-	+	+
	**313(2)**	-	-	+	+
	**331M(3)**	-	-	+	+
	**438M(1)**	-	-	+	+
**CCO**	**298M(2)**	-	-	+	+
	**313(2)**	-	-	+	+
	**331M(3)**	-	-	+	+
**EPC**	**298M(2)**	-	+	+	+

*Summary table of extracts showing toxicity. All other extracts were nontoxic at all tested extract concentrations.

- = no toxic effects observed; + = toxic effects observed

Extract samples available in sufficient amounts were also tested for their cytotoxicities to CCO and EPC cells (Table 3). Again, the results of these cytotoxicity assays showed that nearly all the tested extracts were nontoxic to CCO and EPC cells at the maximum tested concentration of 100 μg/ml. Extracts 298M(2), 313(2) and 331M(3) were toxic to CCO cells at a dose of 100 μg/ml, with 298M(2) definitively being the most toxic (P < 0.001), followed by 313(2) and 331M(3), which showed an approximately equal toxicity (P < 0.01 and P < 0.005, respectively). These data are consistent with visual observations of cell morphology and presence using a microscope. Therefore, these three extracts were used at a concentration of 25 μg/ml in the latter experiments involving CCO cells. Extract 298M(2) was the only extract found to be cytotoxic to EPC cells. It was extremely cytotoxic, as gross cell death was easily visible with a microscope, even at a concentration of 25 μg/ml. For this reason, this extract was used at a concentration of 12.5 μg/ml in the latter experiments involving EPC cells.

### Viral Attachment/Entry Inhibition

**Figure 1:**
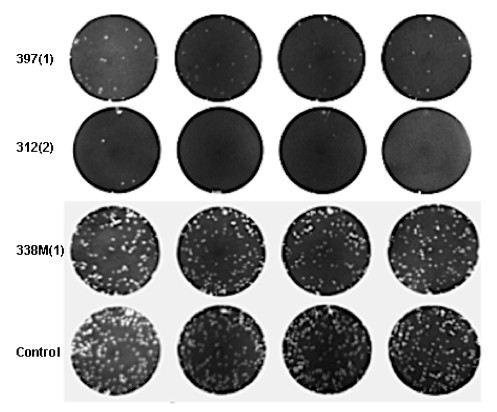
**Representation of viral attachment/entry inhibition by marine extracts**. Viruses (VSV) were pre-incubated with test extract (100 μg/ml). Plates (Vero cells) were infected for one hour, after which plates were allowed to incubate for 24-36 hrs, until adequate plaques were observed. Plates were stained with crystal violet staining and pictures were taken. Plaques were counted and inhibition was determined relative to controls. Row 1: Extract 397(1), showing marked plaque reduction (≥ 80%) relative to the controls; Row 2: Extract 312(2), showing marked plaque reduction (≥ 90%) relative to the controls; Row 3: 338M(1), showing no marked plaque reduction (< 20%) relative to the controls; Row 4: Control of 0.1% DMSO.

Since little is known about the antiviral nature of these marine extracts at the beginning of these experiments, these extracts were first tested for their ability to block viral attachment/entry into the cells. These twenty extracts exhibited different levels of inhibitory effect on viral plaque formation (Table 2, Figure 1). Approximately 14 extracts showed different levels of antiviral impact against HSV-1 in Vero cells (Table 2): three [162M(4), 258M(1) and 367M(1)' possessed high antiviral activity (> 90%), seven [298M(2), 312(2), 313(2), 438M(1), 475(1), 476(1) and 495M(1)' produced moderate inhibitory effects (≥ 50%) and another four [185M(4), 328(2), 331M(3) and 338M(1)' produced slight inhibitory effects (≥ 20%), while the other 6 showed no effect.

The tested extracts also showed varying levels of antiviral impact against VSV in Vero cells (Table 2): five extracts [219M(3), 312(2), 313(2), 328(2) and 367M(1)' showed high antiviral activity (> 80%), while eight other extracts [162M(2), 185M(4), 258M(1), 298M(2), 397(1), 460(1), 475(1) and 476(1)' showed a moderate antiviral effect (≥ 50%). Extract 495M(1) showed slight inhibition, with inhibition being observed as viral plaque reductions of 43%, while the other 6 showed no antiviral effect (< 20%).

Remaining available extracts were tested in CCO cells to determine if they possessed any inhibitory effects towards marine herpes virus CCV (Table 2). Experimental results show that four extracts [298M(2), 313(2), 331M(2) and 397M(1)' had high inhibitory effects against CCV in CCO cells (> 90%). Extract 495M(1) showed moderately high antiviral potential against CCV, with ~90% inhibition, while extracts 388(1) and 397(1) showed moderate antiviral activity, with ~70% inhibition. Extract 162M(4) showed slight antiviral activity (approximately 40% inhibition). The other tested extracts showed no apparent antiviral activities (< 20%).

Remaining available extracts were also tested in EPC cells to determine if they possessed any inhibitory effects towards marine rhabdovirus SHRV (Table 2). Experimental results show that extract 313(2) was the only extract producing high antiviral activity against SHRV in EPC cells, with an inhibition of > 90%. Three other extracts [397M(1), 298M(2) and 331M(2)' showed moderate to low inhibitory properties towards SHRV in EPC cells, with inhibition being ~50%, ~30%, and ~25%, respectively. All other tested extracts showed no apparent inhibition.

### Viral Replication Inhibition

**Figure 2:**
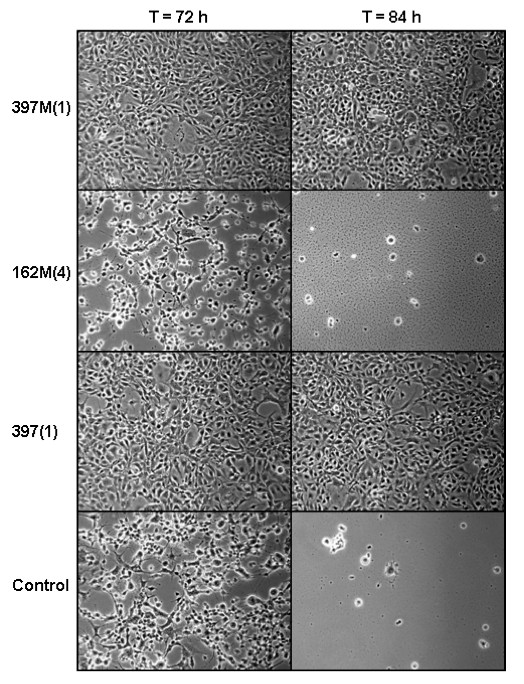
**Representation of viral replication inhibition by marine extracts**. Cells (CCO) were seeded into TC-12.5 cm^2 ^flasks and then infected with virus (CCV) at an MOI of 0.1. Following a 1-hr incubation, media was completely removed and infected cultures were subsequently incubated for approximately 3 days with 2.5 ml/flask of media containing extracts (100 μg/ml). Pictures were taken to track the progression of viral-induced CPE. As shown, pictures were taken at 72 and 84 hours post-infection. Extracts 397(1) and 397M(1) show > 90% viral inhibition, under the parameters of the experiment, relative to the control. Extract 162M(4) shows no inhibition relative to the control.

In addition to viral attachment/entry, marine extracts potentially possess other means of virus inhibition, such as affecting viral replication after the cell is infected. Therefore, an additional set of experiments were performed to determine if these extracts can inhibit virus replication. Results from the viral replication inhibition experiments showed different patterns of antiviral activity, under the described conditions (Figure 2). Extract 298M(2) was the only extract showing antiviral potential against HSV-1. Extract 298M(2) mediated HSV-1 replication within 24 hours post-infection and this antiviral effect was evident throughout the duration of the experiment. At 72 hour post-infection, extract 298M(2) still showed signs of significant viral inhibition, which was visible in the reduction on CPE. Extracts 162M(4), 185M(4) and 397(1) showed signs of viral inhibition against HSV-1 within 24 hours post-infection, however these effects were not present at 72 hours post-infection. Extract 495M(1) showed inhibition against both HSV-1 and VSV within 24 hours post-infection. This effect was not present at the final experimental time-points and any inhibition found was negligible relative to the controls. All other tested extracts were found to possess negligible inhibitive properties against both HSV-1 and VSV. This observation was based on CPE tracking, as well as the production of infectious viruses.

Extracts 331M(2) and 397M(1) showed significantly high inhibition of CCV replication throughout the duration of the experiment, as determined by both reduced CPE and virus production. Extracts 298M(2) and 397(1) showed significantly high inhibition of CCV replication in CCO up to 48 hr post-infection, which decreased slightly by 84 hr post-infection. All remaining extracts tested against CCV in CCO cells were determined to present no significant inhibition (P > 0.05). Viral titers and CPE determined for the remaining extracts were comparable to the control. For SHRV, only extracts 397(1) and 397M(1) showed signs of inhibition under these experimental conditions. At 48 hr post-infection, 100% virus-induced CPE appeared in the control cells, as well as in cultures treated with all other extracts, The cultures treated with extracts 397(1) and 397M(1) showed markedly reduced CPE (25-40%). These results were confirmed by testing culture supernatants for viral titer.

## Discussion

Viral infections are the cause of many human and animal diseases that have tremendous economic impacts. The limited availability of antiviral measures, along with the appearance of new virus types and drug-resistance viral strains, have led scientists to expand their search for novel drug candidates, recently turning back to nature. The marine environment represents an almost inexhaustible resource for antiviral drug leads, as oceans encompass majority of the earth and its highly varying dynamic nature has produce a wide range of organisms that possess unique structures and produce distinctive secondary metabolites. In this study, in vitro assays were established and employed to screen 20 marine microorganism extracts for antiviral activity against four viral isolates that are readily available in this laboratory.

To properly test these marine extracts for antiviral activity, highly concentrated starting materials and broad dose-response studies provide the greatest amount of information. However, high concentrations of marine extracts may be toxic to cell cultures. To address this, a set of experimental tests was performed to determine the safe and effective dose of these test extracts for individual cell culture systems. The concentration of 100 μg/ml was chosen as the maximum test concentration because drug-like molecules are typically sought to have the desired effect at concentration less than or equal to 100 μg/ml [26]. In most drug development cases, drug candidates that require concentration higher than 100 μg/ml are often discarded due to tolerance and cytotoxicity issues, as well as cost effectiveness. Also, because these are extracts and not purified compounds, the active molecule, if any, may be at a very low concentration within the extract and a concentration of 100 μg/ml may allow for any molecule present to produce an antiviral effect. The fact that most extracts remained nontoxic throughout the 3-day experiment was promising. All future experiments would rely on plaque assays that have an incubation time of up to 72 hrs. This time requirement falls well within the range that these extracts were shown to be nontoxic, thus validating the use of these extracts in future experiments that test for antiviral activity.

The extracts were first tested for their ability to block viral attachment/entry into the cells. Viruses were pre-incubated with test extracts at their maximum safe concentration to allow any interactions to take place that may cause the neutralization of virus infectivity, possibly by binding to and blocking the virus itself from adhering to cells, or by blocking the cellular receptors that are utilized by the virus to enter the cells. This reduction of viral infectivity was determined by a reduced number of viral plaque formations relative to controls containing only virus (Figure 1). The initial evaluation of these marine-extract specimens demonstrated that some of these extracts have antiviral potential.

Results from these tests showed that these extracts provided a significantly higher amount of inhibition of VSV plaque formation than HSV-1 plaque formation, in Vero cells. This phenomenon may be attributed to the nature of the envelope proteins of rhabdoviruses. When comparing the inhibitive natures of these extracts, it was found that the extracts appear to show no consistent pattern of inhibition (Table 2). For HSV-1, the mammalian herpes virus, many of the extracts were not strongly preventative of viral entry or infectivity. On the other hand, for the marine herpes virus CCV, many extracts showed inhibitive properties and a few were extremely potent. Ecological pressures, such as competition for space, predation, symbiosis and tide variations, throughout thousands of years, originated the biosynthesis of complex secondary metabolites marine microorganisms, which in turn, allowed their adaptation to a competitive and hostile environment [3]. This could lead to speculation that any viral inhibitive properties possessed by these marine microorganism extracts would be more suited against marine viruses. Unfortunately, this proposal is negated by good-to-excellent anti-viral properties of these marine microorganism extracts against the mammalian rhabdovirus VSV. Many of the tested extracts demonstrated excellent VSV inhibition, but very few (in fact, only one) extracts were effective against the marine rhabdovirus SHRV. A more likely explanation is that the results obtained herein are due to the specific nature of the antiviral mechanisms, producing differential toxicity to individual viruses.

Host cell composition and the factors present in each individual cell culture system may play a role in the effectiveness of each extract's inhibition. The cellular receptors available for viral attachment and entry may differ greatly between each cell type. One may contain a virus-specific receptor that the components contained in an extract can possibly bind to and block, while another cell culture system may possess this same receptor along with additional receptors with redundant functionality that might result in no apparent viral inhibition. Another contributing factor may be each cell line's differential porosity to each extract's components. One extract's antiviral element may be able to get into a specific cell line easier than another, thus possibly producing some replication inhibition in one cell line and not the other. Further testing is needed to identify any of these contributing or limiting factors. Future tests can be specifically designed for a specific virus and host organism, thus eliminating any of these concerns.

In addition to viral attachment/entry, marine extracts potentially possess other means of virus inhibition, such as affecting viral replication after the cell has been infected. It was observed that some extracts showed varying degrees of viral inhibition for HSV-1 during early replication; however this did not last in later stages of infection. It is unknown at this time whether or not the early inhibitory effects are transient due to the active molecule being metabolized or degraded in culture, or if the viral load increased to such an extent that the active molecule was rendered ineffective. For CCV, extracts 331M(2) and 397M(1) showed significantly high inhibition of CCV replication throughout the duration of the experiment. This closely resembled the results from the attachment/entry inhibition assays. This significant inhibition was seen in the CPE tracking, as well as the plaques assay results. These results may be reflecting the extracts ability to prevent re-infection of the cells by blocking the virus released into the media, however this is unknown at this time. It appears that extract 298M(2) shows promise as a potential inhibitor of herpes virus replication, as it show inhibitive properties to HSV-1 and CCV.

Due to the small-scale of this initial study, there did not appear to be strong correlations between the amount of viral inhibition and the extract's organism of origin, however some general inferences were gained. For instance, extracts 312(2) (*Bacillariophyceae cf. Odontella sp*.), 313(2) (*Bacillariophyceae Amphora sp*.) and 328(2) (*Bacillariophyceae cf. Odontella sp*.) all showed highly inhibitive properties for both HSV-1 and VSV viruses, so one may infer that the marine diatom has some general antiviral properties that are common across diatom subspecies. This statement is tentative and will require more examination to corroborate. Extract 258M(1) from *Cyanobacter sp*. also showed very high levels of inhibition for both HSV-1 and VSV. By this same reasoning, one might infer that cyanobacteria hold some general antiviral properties. Other extracts (162M(4), 298M(2), and 367M(1)) come from as-yet unidentified bacterial origins, although they too showed high levels of general antiviral activity for both HSV-1 and VSV. It will be interesting to see if these extracts also come from *Bacillariophyceae*, *Cyanobacter *or another genus or species.

There was also no detectable correlation between significant viral inhibition due to active factor(s) that are secreted (media extracts) or cell-based (whole organism extracts). An equivalent number of cell-and supernatant-derived extracts were tested for their inhibitory effects. Both media and cell extracts alike showed varying levels of inhibition. Equal numbers of cell-derived and supernatant-derived extracts were shown to produce high to moderate levels of viral inhibition, therefore these data do not elucidate whether or not the precise molecules within the extract that possess the antiviral properties. Further studies, using direct comparison of the media extracts from cultured marine microorganisms alongside whole-cell extracts of each organism, will be important for determining the location and differential production of soluble secreted or intracellular antiviral factors.

There were likewise no correlations between the inhibition of viral plaque formation and cytotoxic activity. There were several examples of compounds that were found to be cytotoxic and also inhibited virus plaque formation (298M(2), 313(2), 331M(3) and 438M(1)). These compounds would be less attractive targets for further development as antivirals unless they can be modified to reduce their non-specific cytotoxicity. A contributing factor to underlying cytotoxicity may be the physical state of the starting extract. Most extracts were liquids that ranged in color from a light-yellow to a dark yellow, and even to light brown, with no particulate matter. However, there were some extracts, namely 313(2), 328(2), 460(1) and 491(1), that had distinct physical properties. These extracts were all cell-pellet extracted and their consistencies were more viscous and gelatinous than the other extracts, although they too did not contain particulate matter. One notable exception was 313(2), a dark brown and gelatinous extract containing a substantial amount of particulate matter. Extracts of this nature may somehow interfere with cellular stability or simply creates a hostile environment for cellular growth, producing toxicity. In parallel, the same may be true about its antiviral effects. Perhaps viscous extracts interact directly with the virus or cells, by simply creating a physical barrier that prevents viral attachment. Further testing is needed to elucidate any answers.

Taken together, the observed inhibition does not seem sufficient to suggest the application of these extracts as treatments of established viral infection. Instead, these extracts may have potential use in prophylaxis to prevent infection, as well as preventing the spread of infection, due to the high level of inhibition displayed in the attachment/entry inhibition assays. This is particularly pertinent in confined marine habitats that can be seeded with the active elements of these extracts in hopes of preventing the spread of viral diseases and decreasing mortality.

Future studies can be focused on the isolation of the active elements contained in these extracts. If the individual chemical components of the extracts can be identified, then study of the exact chemical properties against specific viral genomic or proteomic components will be more convincing in demonstrating direct anti-viral mechanisms. It is also possible that any of the observed antiviral effects resulted from synergy between compounds found within the same extract. Alternatively, fractionation and isolation could have the opposite effect of eliminating any antiviral potential. This is because it is well accepted that natural products are sometimes efficacious due to additive or synergistic action between multiple components within the matrix. Therefore, taking a traditional Pharmaceutical Chemistry approach to isolating individual chemicals may destroy the activity of the complex mixture. In any event, characterization of the antiviral compounds and extracts, and elucidation of their antiviral mechanisms and their parental marine organisms, will be key in the discovery of new compounds to be used as antiviral agents. Isolation, identification and characterization of marine compounds and extracts from marine microorganisms with anti-viral effects presents several potential implications, including the important application as chemotherapeutic and/or prophylactic agents of viral diseases of humans, lower animals and marine animals, particularly in aquaculture and conservation biology applications. The identification, chemical and genetic characterization of the active principle(s) and moieties will facilitate the future application of biotechnological procedures for increased yields and cost-effective production.

## Conclusions

Hawai'i represents a geographical location where biologically useful products can be actively discovered [22]. New classes of organisms with novel characteristics are constantly being discovered within the Hawaiian archipelago. Already, a few purified bioactive compounds and over 2,000 unpurified crude extracts from a variety of marine organisms, including sponges, bacteria and algae, have been prepared. Future studies will have access to these previously established and readily available resources. The tests performed in this study have been optimized and can be performed on a larger scale to establish correlations and trends not seen in this small-scale study. The amount of viruses, host cell culture systems, as well as tested extracts can be greatly expanded to yield more conclusive results. With the knowledge gained from large-scale tests, it may be possible to optimize candidate search parameters of not only readily available extracts, but also the search for new novel organisms to be extracted, saving time and money. Due to the almost infinite amount of organisms that can be examined and taking into consideration the environmental pressures that cause similar organisms to evolve and develop unique physical structures and secondary metabolites, it is reasonable to conclude that discovering novel antiviral drugs from marine microorganisms is feasible and likely to be of considerable value for emerging pharmaceutical needs.

## Competing interests

The authors declare that they have no competing interests.

## Authors' contributions

JY carried out the cytotoxicity assays, the viral attachment/entry inhibition assays and the viral replication inhibition assays, as well as drafted the manuscript. YY participated in the initial experimental tests and data analysis of the study as well as provided useful technical input for assay protocols. RB provided the extracts that were made previously for another study, as well as provided necessary information regarding the origin and preparation of the extracts. HTR provided marine isolates for the cultures and extracts. YL was the principle investigator of this project and designed and conceived of the study, and participated in its coordination, and data analysis and manuscript revision. All authors read and approved the final manuscript.
